# An artificial intelligence method using FDG PET to predict treatment outcome in diffuse large B cell lymphoma patients

**DOI:** 10.1038/s41598-023-40218-1

**Published:** 2023-08-12

**Authors:** Maria C. Ferrández, Sandeep S. V. Golla, Jakoba J. Eertink, Bart M. de Vries, Pieternella J. Lugtenburg, Sanne E. Wiegers, Gerben J. C. Zwezerijnen, Simone Pieplenbosch, Lars Kurch, Andreas Hüttmann, Christine Hanoun, Ulrich Dührsen, Henrica C. W. de Vet, PETRA, Otto S. Hoekstra, Coreline N. Burggraaff, Annelies Bes, Martijn W. Heymans, Yvonne W. S. Jauw, Martine E. D. Chamuleau, Sally F. Barrington, George Mikhaeel, Emanuele Zucca, Luca Ceriani, Robert Carr, Tamás Györke, Sándor Czibor, Stefano Fanti, Lale Kostakoglu, Annika Loft, Martin Hutchings, Sze Ting Lee, Josée M. Zijlstra, Ronald Boellaard

**Affiliations:** 1 Cancer Center Amsterdam, Department of Radiology and Nuclear Medicine, Amsterdam UMC, Vrije Universiteit Amsterdam, Amsterdam UMC, Amsterdam, The Netherlands; 2 Cancer Center Amsterdam, Imaging and Biomarkers, Amsterdam, The Netherlands; 3 Cancer Center Amsterdam, Department of Hematology, Amsterdam UMC, Vrije Universiteit Amsterdam, Amsterdam, The Netherlands; 4 Department of Hematology, Erasmus MC Cancer Institute, University Medical Center Rotterdam, Rotterdam, The Netherlands; 5 Department of Nuclear Medicine, Clinic and Polyclinic for Nuclear Medicine, University of Leipzig, Leipzig, Germany; 6 Department of Hematology, West German Cancer Center, University Hospital Essen, University of Duisburg-Essen, Essen, Germany; 7 Department of Epidemiology and Data Science, Amsterdam Public Health Research Institute, Amsterdam UMC, Vrije Universiteit Amsterdam, Amsterdam, The Netherlands; 8 Department of Methodology, Amsterdam Public Health Research Institute, Methodology, Amsterdam, The Netherlands; 9 King’s College London and Guy’s and St Thomas’ PET Centre, School of Biomedical Engineering and Imaging Sciences, King’s Health Partners, King’s College London, London, UK; 10 Department of Clinical Oncology, Guy’s Cancer Centre and School of Cancer and Pharmaceutical Sciences, King’s College London University, London, UK; 11 SAKK Swiss Group for Clinical Cancer Research, Bern, Switzerland; 12 Department of Oncology, IOSI - Oncology Institute of Southern Switzerland, Universita’ Della Svizzera Italiana, Bellinzona, Switzerland; 13 Department of Nuclear Medicine and PET/CT Centre, Imaging Institute of Southern Switzerland, Universita’ Della Svizzera Italiana, Bellinzona, Switzerland; 14 Guy’s and St. Thomas’ Hospital, King’s College, London, UK; 15 Department of Nuclear Medicine, Semmelweis University, Budapest, Hungary; 16 ScanoMed Medical Diagnostic Research and Training Ltd., Budapest, Hungary; 17 Medical Imaging Centre, Department of Nuclear Medicine, Semmelweis University, Budapest, Hungary; 18 Nuclear Medicine Unit, IRCCS Azienda Ospedaliero-Universitaria di Bologna, Bologna, Italy; 19 Radiology Unit, IRCCS Azienda Ospedaliero-Universitaria di Bologna, Bologna, Italy; 20 Nuclear Medicine, Alma Mater Studiorum, University of Bologna, Bologna, Italy; 21 Department of Radiology and Medical Imaging, University of Virginia, Charlottesville, VA USA; 22 Department of Clinical Physiology and Nuclear Medicine, Rigshospitalet, Copenhagen, Denmark; 23 Department of Haematology, Rigshospitalet, Copenhagen, Denmark; 24 Australasian Association of Nuclear Medicine Specialists, Balmain, NSW Australia

**Keywords:** Cancer imaging, B-cell lymphoma

## Abstract

Convolutional neural networks (CNNs) may improve response prediction in diffuse large B-cell lymphoma (DLBCL). The aim of this study was to investigate the feasibility of a CNN using maximum intensity projection (MIP) images from ^18^F-fluorodeoxyglucose (^18^F-FDG) positron emission tomography (PET) baseline scans to predict the probability of time-to-progression (TTP) within 2 years and compare it with the International Prognostic Index (IPI), i.e. a clinically used score. 296 DLBCL ^18^F-FDG PET/CT baseline scans collected from a prospective clinical trial (HOVON-84) were analysed. Cross-validation was performed using coronal and sagittal MIPs. An external dataset (340 DLBCL patients) was used to validate the model. Association between the probabilities, metabolic tumour volume and Dmax_bulk_ was assessed. Probabilities for PET scans with synthetically removed tumors were also assessed. The CNN provided a 2-year TTP prediction with an area under the curve (AUC) of 0.74, outperforming the IPI-based model (AUC = 0.68). Furthermore, high probabilities (> 0.6) of the original MIPs were considerably decreased after removing the tumours (< 0.4, generally). These findings suggest that MIP-based CNNs are able to predict treatment outcome in DLBCL.

## Introduction

Diffuse large B-cell lymphoma (DLBCL) is an aggressive lymphoid malignancy which originates in the B lymphocytes and accounts for 30% of the total annual diagnoses of Non-Hodgkin lymphoma in western countries^1^. A widely used first-line therapy in DLBCL combines rituximab, cyclophosphamide, doxorubicin, vincristine, and prednisone (R-CHOP). The number of R-CHOP cycles and/or initial usage of more intense chemotherapy regimens initially depends on the primary disease stage and the International Prognostic Index (IPI), which defines a patient's risk profile^2^. IPI score includes age, World Health Organization performance status, Ann Arbor stage, serum lactate dehydrogenase level, and number of extranodal sites of disease. ^18^F-fluorodeoxyglucose (^18^F-FDG) positron emission tomography (PET)—computed tomography (CT) imaging allows highly accurate visualization of DLBCL tumours, which is therefore the essential modality for appropriate staging. Moreover, ^18^F-FDG PET is frequently used as an early outcome prediction marker, since complete metabolic response early throughout (i.e. interim) therapy allows de-escalation of treatment cycles^3^. This interim-PET adaptive treatment approach is increasingly integrated into national recommendations on DLBCL. Despite better identification of low-risk patients at baseline and early during treatment, overall, one-third of DLBCL patients do not respond to first-line treatment or relapse^1^. Therefore, early identification of high-risk patients is important as patients might benefit from a more tailored treatment strategy.

Quantitative parameters extracted from ^18^F-FDG PET/CT scans provide insight into the tumour characteristics. From these parameters, metabolic tumour volume (MTV) has been repeatedly reported as a promising prognostic factor in DLBCL^4–6^. The inclusion of dissemination features like the maximal distance between the largest lesion and any other lesion (Dmax_bulk_), in combination with MTV, has further improved risk stratification of patients^5^. To obtain these parameters, tumour segmentation requires user interaction for each ^18^F-FDG PET/CT scan which can be time consuming and depends on the observers interpretation. The implementation of artificial intelligence (AI) and convolutional neural networks (CNNs) might be able to reduce and/or replace these tasks. CNNs can extract high-level features from multi-dimensional input data (i.e. images). In oncology, CNNs are already being investigated to automate different medical image classification tasks: diagnostics^7^, tumour delineation and segmentation^8–10^, extraction of PET features surrogates^11^ and disease progression and/or treatment outcome prediction^12,13^. Two of the main drawbacks of CNNs are the computational expense they entail and the high complexity of its extracted features, especially when it comes to 3D image analysis such as with PET/CT scans. Alternatively, maximum intensity projections (MIPs) of ^18^F-FDG PET/CT scans can be used as 2D inputs for the CNN, decreasing data dimension and complexity and, thereby, decreasing the computational load and cost^14,15^.

The aim of this study was to investigate the feasibility of a CNN for the prediction of 2-year time-to-progression (TTP) in DLBCL patients using MIP images derived from ^18^F-FDG PET/CT baseline scans. The models outcome is a binary prediction given by the probability of TTP longer than 2 years P(TTP0) or TTP shorter than 2 years P(TTP1), where TTP1 indicates an increased risk of tumour progression for the patient. TTP0 may indicate absence of tumour progression or absence of recurrence.

## Materials and methods

### Datasets

In this study we used two different datasets of baseline DLBCL ^18^F-FDG PET/CT scans: the HOVON-84 dataset^16^ was used to train the CNN model whereas the PETAL dataset^17^ was used as an external validation of the models performance. All patients from both datasets provided written consent for the use of their data. After correction for IPI, there were no significant differences in survival between the PETAL and HOVON-84 study^18^. Both studies were approved by institutional review boards and all included patients provided informed consent. The use of all data within the PETRA imaging database has been approved by the institutional review board of the VU University Medical Center (JR/20140414).

#### HOVON-84

Three hundred seventy-three DLBCL patients who underwent baseline ^18^F-FDG PET/CT from the multicenter HOVON-84 trial (EudraCT, 2006-005,174-42) were included in this study. The main inclusion/exclusion criteria from this trial can be found elsewhere^16^. From these, 317 diagnosed DLBCL patients were included in this analysis. Missing essential DICOM (Digital Imaging and Communication in Medicine) information and incomplete whole-body scans were the main reason for exclusion. Furthermore, 7 patients were lost to follow-up within 2 years and 14 other patients died within 2 years of unrelated reasons. This led to a total of 296 DLBCL patients included in the study. Of which, 244 were classified as TTP0 and 52 as TTP1. In this paper, we used the exact same data as previously published by Eertink et al.^4^ to allow for direct comparison of our results.

#### PETAL

The external validation was performed using diagnosed DLBCL patients from the multicenter PETAL trial (EudraCT 2006-001641-33) who underwent baseline ^18^F-FDG PET/CT. The eligibility for the PETAL trial is described elsewhere^17^. Initially, the trial consisted of 1098 PETAL patients. Reasons to exclude patients were as follows: diagnosis other than DLBCL, incomplete scans or with artefacts or missing DICOM information. This led to a total of 395 DLBCL patients with associated ^18^F-FDG PET/CT baselines scans available for this study. Moreover, 12 underwent a different treatment to R-CHOP, 24 patients were lost for follow-up within 2 years, and 19 died without progression. This led to a total of 340 patients. From these, 279 were classified as TTP0 and 61 as TTP1. The exact same data were used as in Eertink et al.^4,19^, so that our results can be compared with recently published segmentation based approaches.

#### Quality control of scans

The participating sites provided the scans in DICOM format. The scans were subsequently anonymised. For QC we used criteria described by EANM guidelines: mean standardized uptake value (SUVmean) of the liver should be between 1.3 and 3.0 and the plasma glucose level lower than 11 mmol/L^3^. The QC criteria are described in detail elsewhere^4^.

### Maximum intensity projections

**Figure 1:**
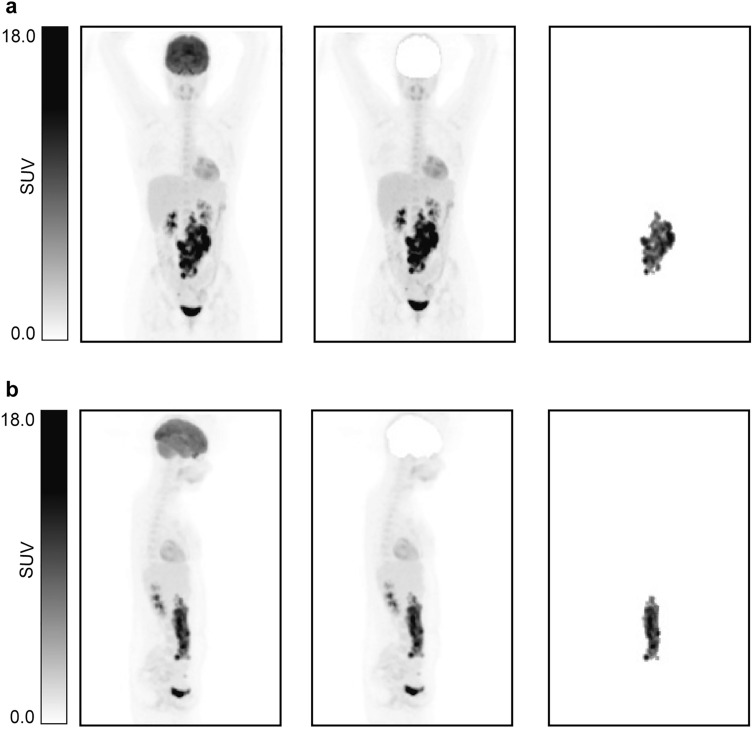
Illustration of the different MIPs implemented in this study. (**a**) Coronal view. (**b**) Sagittal view. From left to right: MIP, MIP without brain and lesion MIP.

The ACCURATE tool was used to obtain the so-called lesion masks which are the images that contain only the lymphoma tumour(s) segmentation^20^. The segmentation of the tumours was performed using a SUV threshold of 4.0. This was found to be the preferred segmentation threshold, as shown by Barrington et al.^21^. Any physiological uptake adjacent to tumours was manually deleted. The conversion to MIP images was performed using a preprocessing tool developed in Interactive Data Language (IDL®, NV5 Geospatial Solutions, Inc). This tool generates coronal and sagittal MIPs with size 275 × 200 × 1 and a pixel size of 4 × 4 mm. MIPs were generated for lesion MIPs (MIPs containing only tumours) and for the complete PET scans. Examples of these coronal and sagittal MIPs can be found in Fig. 1.The MIPs were normalized by a fixed maximum intensity value (SUV = 40). This was selected based on the maximum tumour intensity value found across the scans. The values above this maximum were truncated to avoid normalization to be driven by the SUV value of high uptake organs such as the bladder.

### Data sampling scheme

**Figure 2:**
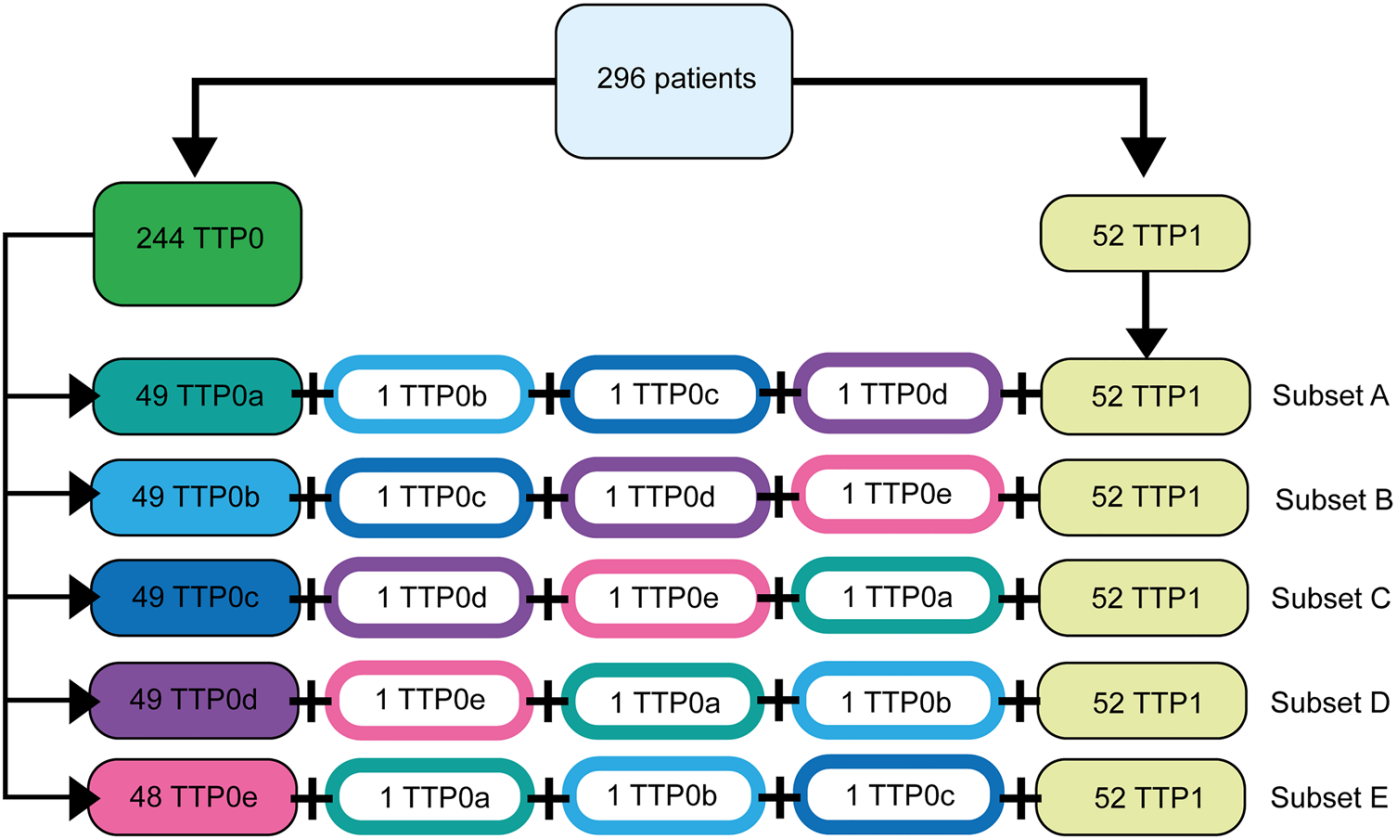
Data sampling scheme. Diagram representation of the five data subsets, with four subsets (A–D) consisting of 52 stratified TTP0 subjects and all 52 TTP1 subjects. One of the five subsets (subset E) contained 51 stratified TTP0 and all 52 TTP1 subjects.

Since the training dataset classes were highly unbalanced (244 TTP0 vs 52 TTP1), we applied a data sampling scheme were the TTP0s were divided into 5 equally stratified data subsets: 4 subsets of 49 patients (subsets A-D) and 1 subset of 48 patients (subset E). Additionally, 3 randomly selected TTP0 patients belonging to different subsets, were added to each of these subsets in order to achieve a total of 52 TTP0s per subset (subset E with 51 TTP0s), which matched the total number of TTP1s. Eventually, each subset contained a total of 104 patients with a prevalence of 50% for each class (subset E with 103 patients). The details of this scheme can be found in Fig. 2. Each subset (A to E) was trained using a fivefold cross validation (for each cross validation run, the data was split into two sets: training set (80%) and internal validation set (20%)).

### Convolutional neural network

**Figure 3:**
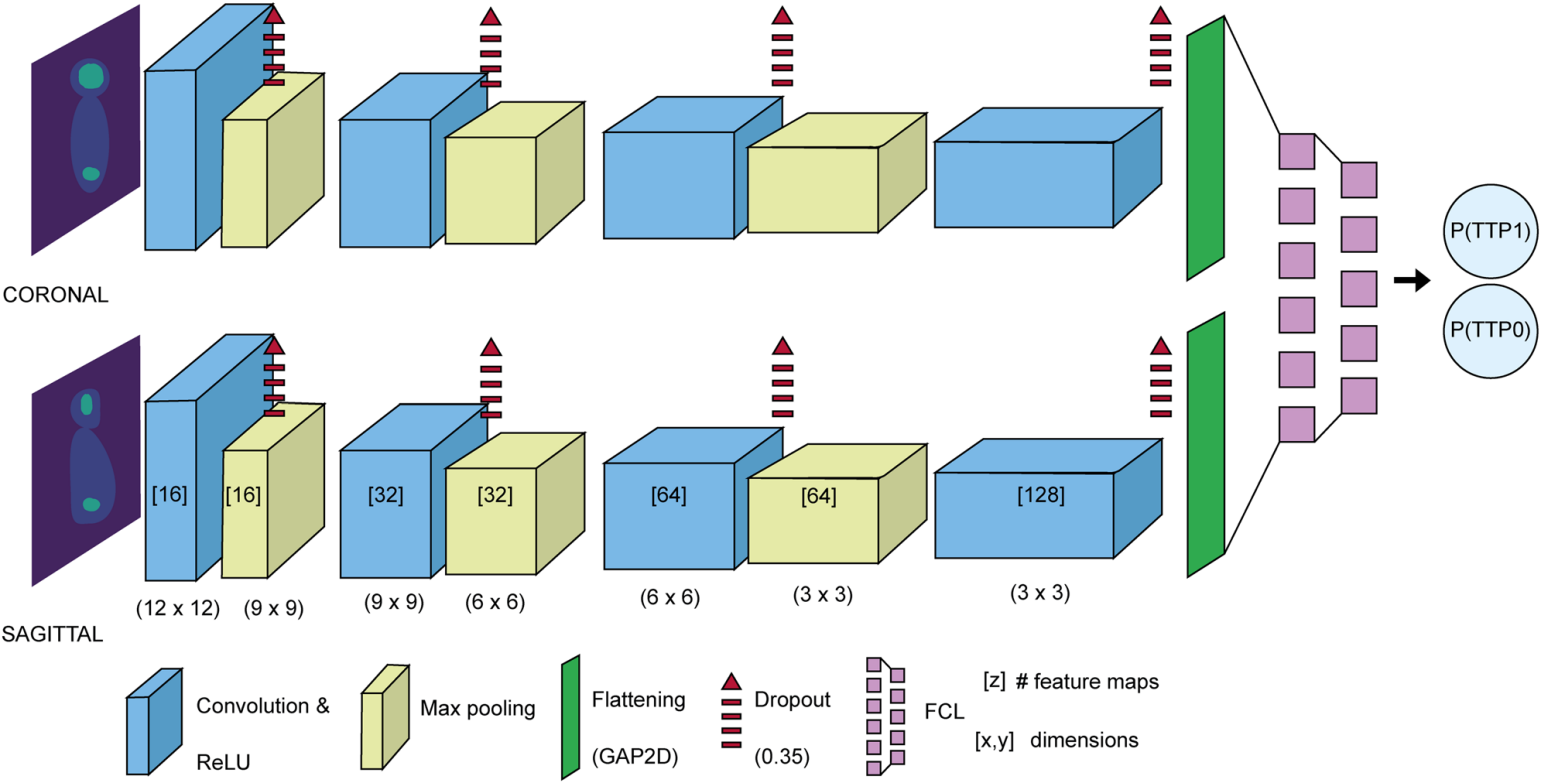
CNN architecture. In the convolution layers, the number of feature maps is shown, followed by the size of these matrices. The max pooling layers are depicted with the feature detector dimensions. A spatial dropout and the ReLU activation function were applied to each convolution layer. The model was compiled using the Adam optimizer, with LRt = 0.00005 and DR = 0.000001. Coronal and sagittal outputs are concatenated in the dense layer or fully connected layer (FCL).

The CNN consists of two branches, one receives the coronal MIP as input and the other one receives the sagittal MIP as input, which are merged as a last step to yield the final prediction. Coronal and sagittal MIPs are analysed independently but in parallel by an identical multi-layer architecture. The CNN design consists of 4 convolution layers, each one of these are followed by a max pooling layer. In a CNN, the convolution layer uses different filters over the image to extract low level features (e.g. edges, gradients) in earlier layers and high level features in deeper layers. In our CNN, the feature maps are doubled at each convolution layer, starting at 16 in the first layer and going up to 128 in the last layer, and their dimensions continuously decrease by (3,3). In each convolution layer the rectified linear unit (ReLU) activation function is applied. After each convolution layer, a dropout of 0.35 is applied, indicating that 35% of the network nodes and connections are randomly dropped from the CNN in order to prevent overfitting. Right after the dropout, a MaxPooling layer was implemented. The MaxPooling layer acts as the dimensionality reduction layer. There are 3 Maxpooling layers in our CNN, each of these with feature map sizes of (3,3), (3,3) and (2,2). After the last convolution and SpatialDropout layer, the CNN is connected to a GlobalAveragePooling2D (GAP2D) layer also known as ‘flattening’ layer. This layer ‘flattens’ the output from the convolution layers into a less complex shape (i.e. a 2D tensor). The coronal and sagittal outputs are then concatenated at the final dense layer or fully connected layer (FCL) which generates an output for two different classes: TTP0 and TTP1. A softmax function is introduced to generate a probability for each of these classes, which is the final CNN output the probability of TTP longer than 2 years, P(TTP0), and the probability of TTP shorter than 2 years, P(TTP1) both add up to 1. The classifier was compiled using the Adam optimizer with a learning rate (LRt) of 0.00005 and a decay rate (DR) of 0.000001. The CNN structure is illustrated in Fig. 3.

In this study we trained the model following 3 different training schemes. These are illustrated in Supplemental Figure 1: training based on (1) only-lesion MIPs (Lesion MIP CNN); (2) lesion MIPs and regular MIPs (MIP CNN); (3) lesion MIPs and MIPs after removal of the brain, brain removed MIPs (BR-MIP CNN). The network architecture is kept the same. This approach was followed in order to explore if the model could be trained to recognize pathological patterns.

The lesion MIP CNN uses only the lesion MIPs as input (Fig. 1). These MIPs contain only the information and intensity of the tumours. The lesions MIPs of both coronal and sagittal views were used to train and validate the model for 200 epochs.

The MIP CNN uses both lesion MIPs and MIPs for the training of the model. The lesion masks contain only information of the tumours but not the intensity values. The training of the MIP CNN consisted in two subsequent steps. Firstly, the lesions masks of both coronal and sagittal MIPs were used to train and validate the model for 200 epochs. In the second step, the pre-trained model on the lesion masks was re-trained and re-validated for another 300 epochs, this time using the regular coronal and sagittal MIP images instead. The same patients were used for training of the two steps.

The BR-MIP CNN follows the same training process as the MIP CNN but instead of the original MIPs, it uses MIPs without brain (Fig. 1). MIP brain removal was performed in order to provide greater consistency across the dataset since not all scans included the head. The process of removing the brains is described in detail in Supplemental Material 1^22^.

In the case of the MIP CNN and the BR-MIP CNN, the classifier required only the MIP images to make the predictions. The idea behind these two CNNs was to generate a classifier where the prediction is free of the observer-dependent tumour segmentation.

All models were implemented using Python version 3.9.16, Keras version 2.10.0 and Tensorflow library version 2.10.0.

### Plausibility of the CNN

To better understand the CNN predictions, we further investigated the output of the model by exploring the association between P(TTP1) and two PET extracted features: MTV and Dmax_bulk_ since both have shown potential as prognostic markers in DLBCL^4,5,19,23^. The process to extract PET features has been explained in previous studies^4^. Moreover, we synthetically removed the tumours from the MIP images to simulate tumour-free data and evaluated the CNN predictions on this data. The tumours were masked using the lesion MIPs generated using the in-house built preprocessing tool. The voxel values corresponding to the tumours were replaced by an average of the voxel intensities excluding the background. This process is shown in Supplemental Figure 2.

To facilitate representation, the TTP1 probabilities obtained through the CNN were calibrated by performing a logistic regression fit with the probabilities as input and the TTP0/TTP1 labels as outcome^24^. The obtained regression fit coefficients were then applied to the CNN TTP1 probabilities generated after removing the tumours in order to accordingly calibrate the tumour-free MIPs CNN TTP1 probabilities.

### Statistical analysis

The receiver operating characteristic (ROC) and the area under the curve (AUC) were used to evaluate the CNN performance. During training, the fold with the highest cross validated (CV-)AUC across the 5 folds was preserved. See Supplemental Material 2 for more details^25^. The PETAL dataset was used to externally validate the CNN model. This performance evaluation process was performed for the lesion MIP CNN, MIP CNN and BR-MIP CNN. The cut-off value to determine sensitivity and specificity for every model was set to 0.5. AUCs were statistically compared using the Delong test to assess the performance of different CNN models to that of an IPI-based prediction model^26^. This IPI model defines patients with risk factor of 4 or higher as high-risk patients (i.e. TTP1)^4^. The association of the TTP1 probabilities and the PET-extracted features (MTV and Dmax_bulk_) was assessed using Pearson’s correlation coefficient.

### Ethical approval

All individual participants included in the study gave written informed consent to participate in the study. The HOVON-84 study was approved by the institutional review board of the Erasmus MC (2007-055) and was performed in accordance with the ethical standards as laid down in the 1964 Declaration of Helsinki and its later amendments or comparable ethical standards. The PETAL study was approved by the Federal Institute for Drugs and Medical Devices and the ethics committees of all participating sites (University Hospital Essen and Deutsche Krebshilfe; ClinicalTrials.gov NCT00554164).

## Results

A summary of the characteristics of the datasets can be found in Supplemental Table 1.

**Table 1:** Cross-validation (± SD) of AUC, sensitivity, and specificity for training and internal validation (HOVON-84 dataset) for the model associated with subset C.

CNN	CV-AUC (SD)		Sensitivity (SD)		Specificity (SD)	
	Training	Validation	Training	Validation	Training	Validation
Lesion MIP	0.81 (0.02)	0.75 (0.07)	0.69 (0.06)	0.63 (0.19)	0.76 (0.02)	0.73 (0.08)
MIP	0.79 (0.03)	0.70 (0.06)	0.76 (0.04)	0.75 (0.08)	0.64 (0.06)	0.55 (0.10)
BR-MIP	0.77 (0.09)	0.72 (0.11)	0.73 (0.17)	0.71 (0.14)	0.69 (0.11)	0.63 (0.19)

*AUC* area under the curve, *SD* standard deviation.

### Internal validation

**Figure 4:**
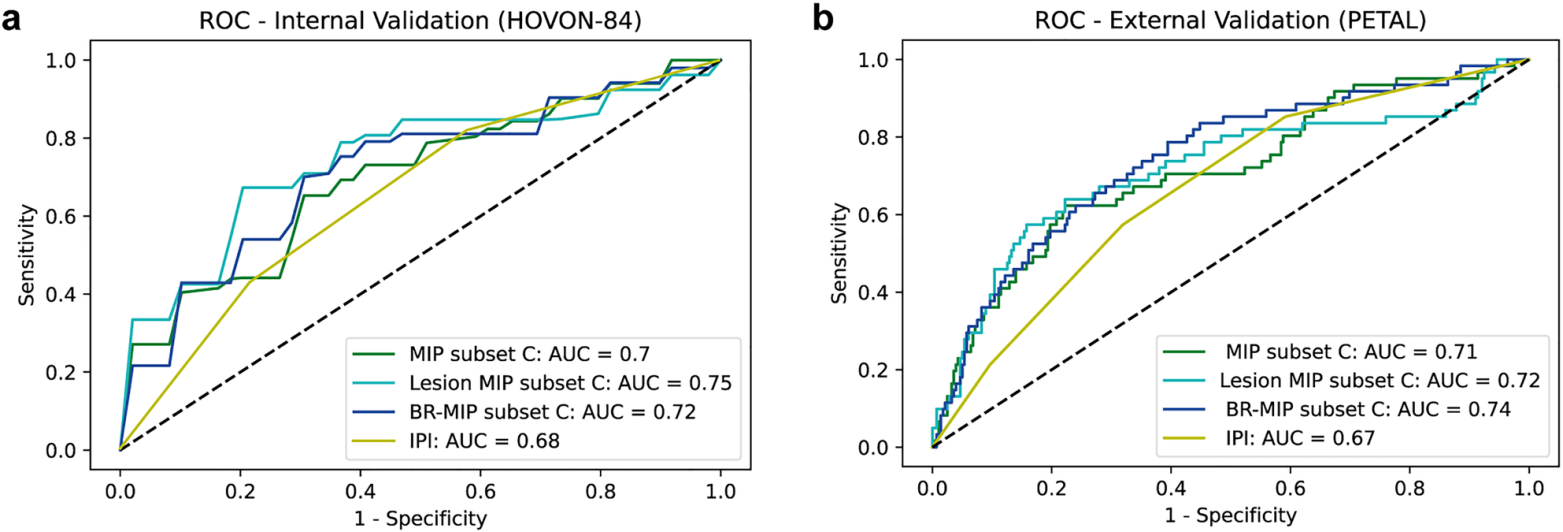
Receiver Operator Curves. (**a**) ROC and AUC for internal validation performed on HOVON-84 dataset for the model trained on subset C (following fivefold cross validation). (**b**) ROC and AUC for external validation performed on PETAL dataset using the model trained on subset C. For reference, a model without any predictive performance is depicted (AUC = 0.5).

The results for the fivefold CV for the 5 data subsets (A–E) can be found in the Supplemental Tables 2–4. The average performance of the models (associated with each subset) can be found in Supplemental Figure 3a. The model trained with subset C was best performing in all cases (Supplemental Figure 4). The AUCs for the model trained on subset C for Lesion MIP CNN, MIP CNN and BR-MIP CNN are illustrated in Fig. 4a and the CV-AUCs are given in Table 1. These values are comparable or better to the ones obtained from the IPI prediction model (AUC was reported as 0.68 for the HOVON-84 dataset)^4^. Delong test showed statistical significant differences between the AUC curves for the BR-MIP CNN and the IPI prediction model (*p* value = 0.015).

### External validation

The PETAL dataset was used to externally validate the performance of the CNNs. The average performance of the models (associated with each subset) can be found in Supplemental Figure 3b. The model trained with subset C was again the best performing trained model (Supplemental Figure 5). The ROC plot with the corresponding AUC values for each CNN are shown in Fig. 4b. A summary of the performance of the 3 CNNs is given in Table 2. The BR-MIP CNN outperformed the IPI model with an AUC of 0.67, sensitivity = 0.57 and specificity = 0.68 (Delong test, p-value = 0.035). We provided some examples of the BR-MIP CNN predictions in Supplemental Figure 6.

**Table 2:** AUC, sensitivity and specificity for external validation data (PETAL dataset) for the model associated with subset C.

CNN	AUC	Sensitivity	Specificity
Lesion MIP	0.72	0.59	0.8
MIP	0.71	0.62	0.72
BR-MIP	0.74	0.54	0.81

*AUC* area under the curve.

### Plausibility of the CNN

**Figure 5:**
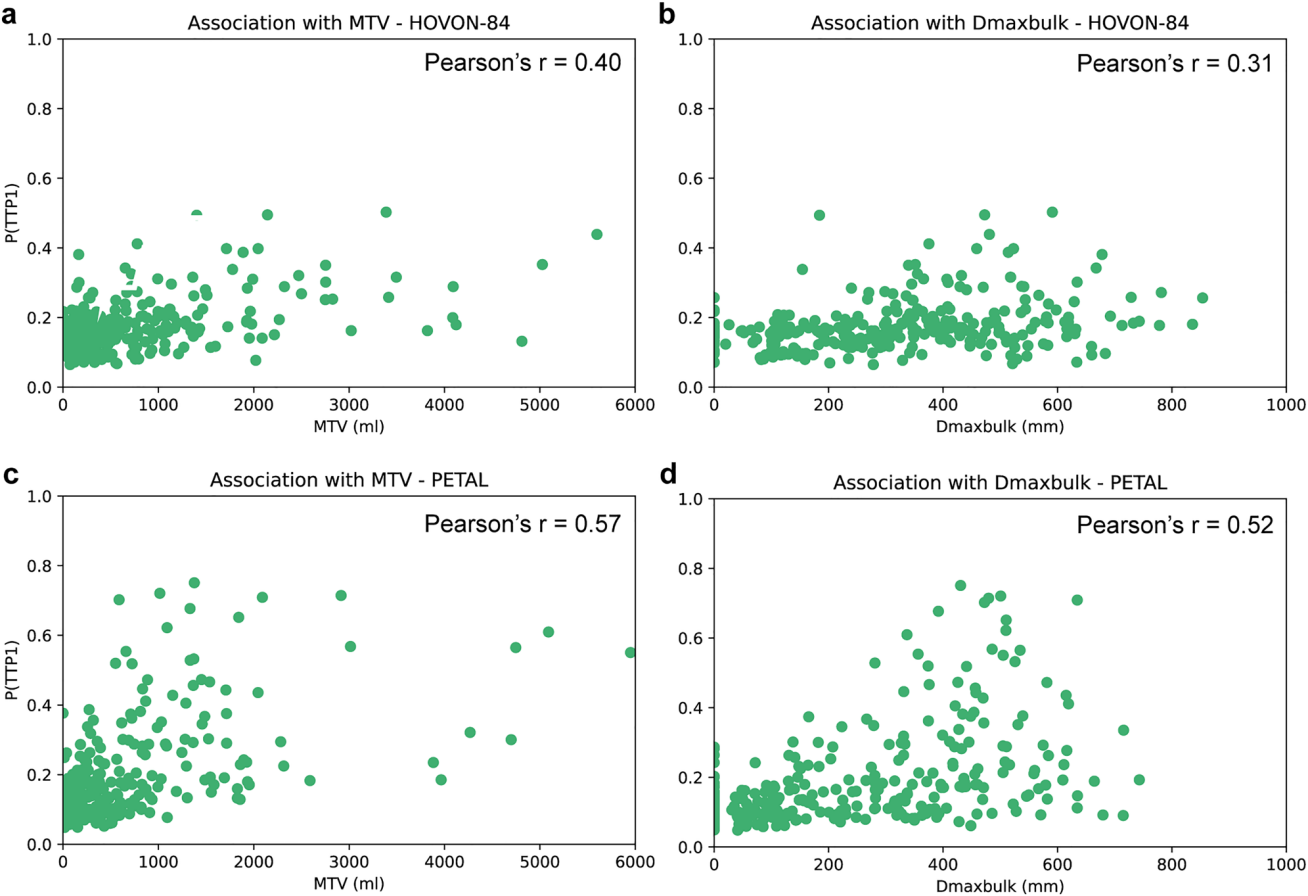
Association between TTP1 probabilities and PET features: (**a, b**) HOVON84 TTP1 probabilities for MTV and Dmax_bulk_ respectively. (**c, d**) PETAL TTP1 probabilities for MTV and Dmax_bulk_ respectively.

The BR-MIP CNN (trained using subset C) was used to further investigate the feasibility of the model for TTP prediction. A moderate association for MTV with P(TTP1) and a weak association for Dmax_bulk_ with P(TTP1) was found for HOVON-84 (Fig. 5a, b) and a moderate association for both MTV and Dmax_bulk_ with P(TTP1) was found for PETAL (Fig. 5c,d). In all scenarios, higher P(TTP1) seemed to be related to higher MTV and Dmax_bulk_ values. These features have been previously reported as promising prognostic factors in DLBCL^4–6^.

**Figure 6:**
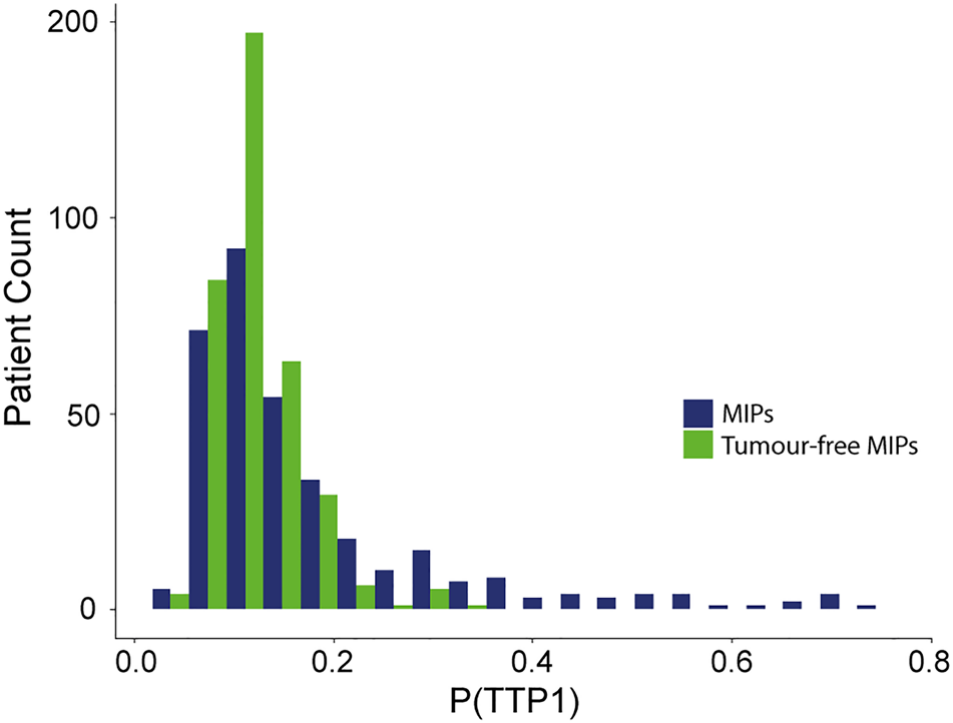
Histogram for the PETAL TTP1 probabilities. Probabilities for MIPs shown in dark blue and probabilities after removing the tumours (i.e. tumour-free MIPs) shown in green.

After generating new probabilities for the tumour-free MIPs in the PETAL dataset, we found that these were generally lower when compared to the initial probabilities obtained from the images with tumours. This is the case, specially, for probabilities over 0.6, which, after tumour removal, were decreased to values below 0.4. Some examples are given in Supplemental Figure 7 for different patients with decreased probabilities after tumour removal. The histogram of P(TTP1)s is shown in Fig. 6 for both datasets: original MIPs (with tumours) and tumour-free MIPs.

## Discussion

In this study, we investigated the feasibility of a CNN model for the prediction of progression after 2 years in DLBCL patients using ^18^F-FDG PET/CT MIP images. Our model was internally (HOVON-84 dataset) and externally validated (PETAL dataset) to assess the performance of the model in a different dataset^18^. Proper external validation is one of the main limitations seen across AI studies^27,28^. Poor or insufficient validation causes misleading results and limits translation into the clinical setting^29^. In our external validation, the CNN showed an improved predictive performance compared to the IPI model (AUC of 0.74 vs 0.67, Delong test p-value = 0.035). In a recent paper by Westin et al.^30^, it is recommended to assess progression within or after 1-year of the first line treatment. We decided to use 2-year TTP because at the time of this study, it remained clinically relevant^31–34^ but most importantly, to be able to compare our model performance with results seen in previous studies^4,19^.

Features extracted from PET scans are currently being investigated to predict outcome of DLBCL patients with promising results^4,19,23,33,35^. Mikhaeel et al*.*^23^ recently published the International Metabolic Prognostic Index which included Ann Arbor stage, age and MTV for the prediction of 3-year progression free survival (PFS). Moreover, Eertink et al*.*^4^ developed a model that combined PET-extracted features (including MTV and Dmax_bulk_) with clinical parameters, yielding a CV-AUC of 0.77 for the prediction of 2-year TTP in an internal validation using scans from the HOVON-84 trial.

In addition, there is an increasing interest in the use of CNNs as segmentation tools. Several studies have shown the potential of CNNs for lesion segmentation in lymphoma patients with outstanding results when comparing surrogate PET features to PET features extracted from manually segmented lesions^9,36–38^. These models are easy to understand, especially in a clinical setting. The segmentation of the lesions can be easily inspected visually and allows direct inspection of tumour volume, dissemination and any other extracted feature, that are used for prediction. The potential of segmentation-based CNNs is unquestionable and for this reason we intent to investigate their role in DLBCL treatment outcome prediction in the future and compare these with deep learning based end-to-end methods.

In this study, we aimed to assess the feasibility of a deep learning model that does not rely on segmentation and generates predictions directly from the PET images. It is important to highlight the fact that this study is meant to be a first step in the exploratory analysis of using end-to-end CNNs. However, these models are difficult to use due to their complexity and lack of interpretability^39^. In addition to these challenges, it is not yet known whether end-to-end CNNs could outperform segmentation-based models and/or radiomic models and therefore, the use of segmentation based AI approaches in combination with handcrafted radiomics analysis should also be further explored.

Currently, there are only a few studies which have looked at the applications of end-to-end CNNs. Liu et al.^40^ developed a multi task 3D U-Net for both tumour segmentation and prediction of PFS in DLBCL with outstanding results when compared to radiomic-based models and single task CNNs. Moreover, the use of MIPs for treatment outcome prediction in DLBCL is currently being investigated^15,40,41^. Rebaud et al.^41^ trained a multi-task ranker neural network using coronal MIP images which performed as well as TMTV for DLBCL PFS prediction. However, these studies did not assess the performance of the model in external datasets as indicated in the RELAINCE guidelines^29^. To our best knowledge, this is the first paper to investigate the feasibility of CNNs for ‘deep’ treatment outcome prediction in DLBCL patients using ^18^F-FDG PET/CT MIP images and to investigate its performance in an external dataset.

We trained the CNN in 3 different ways: lesion MIP, MIP and BR-MIP CNN. The BR-MIP CNN was kept for the CNN plausibility analysis as it outperformed the IPI-based model and predicted 2-year TTP with the highest AUC in the external dataset. Moreover, compared to the lesion MIP, BR-MIP CNN does not require prior tumour segmentation to make predictions and, compared to the MIP CNN, BR-MIP model uses MIP images without brain uptake. The main reason to remove the brain is that it brings consistency across the dataset as, in some PET studies, the head was not (fully) included during acquisition.

In order to better understand the output of our model, we investigated whether there was any relation between the CNN outcome probabilities with MTV and Dmax_bulk_, since both of them have prognostic capabilities for DLBCL. Even though a weak association was found for Dmax_bulk_ with HOVON-84 predictions, our results suggests that the CNN is capturing information that might be related to tumour volume and dissemination but also that other image characteristics influence the CNN prediction. Deep learning methods tend to pay more attention to textural features^42^. In this context, conventional PET parameters, although easier to understand, might be missing relevant information for tumour progression. Another technique we used to examine the plausibility of the model is ‘ablating’ the tumours from the MIPs. The decrease in P(TTP1) values suggest that the CNN is indeed mainly using tumour information to make the predictions. Furthermore, when looking at a few examples of the CNN output (Supplemental Figure 6) we found that patients with fewer tumours and lower dissemination are given lower probability values than patients with more tumours and higher dissemination. Even though further analysis is needed, these findings suggest that the model associates tumour dissemination with a higher risk of disease progression.

Regarding the limitations of this study, DLBCL patients can develop lesions near or within the brain which might complicate brain removal. Even after addressing this issue, there were around 1% of patients with truncated lesions which could not be solved. It is therefore important to consider clinician supervision in these cases. Another limitation of this study is the cut-off value used to calculate the sensitivity and specificity, based on the HOVON-84 dataset, which led to certain differences in the PETAL dataset. Slight adjustment of this value might be required to achieve comparable results in terms of sensitivity and specificity in external datasets. Moreover, HOVON-84 trial did not include patients with Ann Arbor stage 1 disease and patients who had central nervous system involvement were also excluded from the trials. The lack of limited stage and DLBCL patients with very poor prognosis could be a potential bias for the model performance and its generalizability. A more extensive external validation is required to assess the generalizability of the model.

As mentioned earlier, end-to-end CNNs are complex and interpretation of results is difficult. In this study we partially addressed this by looking at associations with known PET parameters and analysing tumour intensities contribution. However, these issues make the translation into the clinic challenging. Nevertheless, we believe more research in this field is required to unravel the potential of end-to-end CNNs.

## Conclusion

In this study we introduced a CNN model capable of predicting 2-year TTP in DLBCL patients using ^18^F-FDG PET/CT MIP images as input. The CNN model predicted 2-year TTP in DLBCL patients better than IPI scores. Moreover, it was illustrated that the model prediction is affected by the presence or absence of lesions. Even though further investigations are necessary, our current findings suggest that CNNs using MIPs have potential as outcome prediction models.

### Supplementary Information

Supplementary Information 1.

Supplementary Information 2.

## Data Availability

The code and the model weights will be available as a supplementary zip file upon publication of the manuscript.
