# Supplementary material for: An artificial intelligence method using FDG PET to predict treatment outcome in diffuse large B cell lymphoma patients
Source: Sci Rep. 2023 Aug 12;13:13111. doi: 10.1038/s41598-023-40218-1 (PMC10423266; doi:10.1038/s41598-023-40218-1)
Supplement: Supplementary file 1 — Supplementary Information 1. [file 41598_2023_40218_MOESM1_ESM.zip › final/Installation_manual.docx]

In order to use the scripts contained in this file you will need to install the following:

- python version 3.9.16
- tensorflow version 2.10.0
- keras version 2.10.0

The list of packages/dependencies required for the conda environment to run these scripts can be found in the file *conda_dependencies.txt*
