# Supplementary material for: An artificial intelligence method using FDG PET to predict treatment outcome in diffuse large B cell lymphoma patients
Source: Sci Rep. 2023 Aug 12;13:13111. doi: 10.1038/s41598-023-40218-1 (PMC10423266; doi:10.1038/s41598-023-40218-1)
Supplement: Supplementary file 1 — Supplementary Information 1. [file 41598_2023_40218_MOESM1_ESM.zip › final/Instructions.docx]

This zip file contains the main scripts related to the manuscript with title: **An artificial intelligence method using FDG PET to predict treatment outcome in diffuse large B cell lymphoma patients**

There are 3 folders in this file:

1. SCRIPTS FOLDER

- File name: *CNN_functions.py*

This script contains relevant functions for the prepocessing and preparation of the scans.

- File name: *MIP_generator.py*

This script is a Python version of the preprocessing tool used to generate the MIPs.

- File name: *MIP_preparation.py*

This script is used to preprocess the scans, the vois, generate the MIPs and brain segmentation. It takes the functions from *CNN_functions.py* and *MIP_generator.py*

- File name: *CNN_architecture.py*

This script contains the design and architecture of the CNN.

- File name: *tumor_removal.py*

This script is used to mask the tumor.

1. EXAMPLE DATA FOLDER

This folder contains an example data of one subject. All of the numpy files can be generated from the script *MIP_preparation.py*

- *HO8400077_C0.prj_PET.nii* : PET scan nifty format
- *HO8400077_C0.prj.VOI.nii* : PET scan containing only VOI nifty format
- *HO8400077.npy* : PET scan numpy format
- *HO8400077_VOI.npy* : VOI-only PET scan numpy format
- *HO8400077_cor_MIPnbr.py* : coronal MIP numpy format
- *HO8400077_sag_MIPnbr.py* : sagittal MIP numpy format
- *HO8400077_cor_lesion.npy* : lesion-only coronal MIP numpy format
- *HO8400077_sag_lesion.npy* : lesion-only sagittal MIP numpy format

1. MODEL FOLDER

Here you will find the weights of the final model which can be deployed and used to make predictions.
