# Supplementary material for: An artificial intelligence method using FDG PET to predict treatment outcome in diffuse large B cell lymphoma patients
Source: Sci Rep. 2023 Aug 12;13:13111. doi: 10.1038/s41598-023-40218-1 (PMC10423266; doi:10.1038/s41598-023-40218-1)
Supplement: Supplementary file 2 — Supplementary Information 2. [file 41598_2023_40218_MOESM2_ESM.docx]

**An artificial intelligence method using FDG PET to predict treatment outcome in diffuse large B cell lymphoma patients**

**Authors and Affiliations:** Maria C. Ferrández*^1,2^, Sandeep S. V. Golla^1,2^, Jakoba J. Eertink^2,3^, Bart M. de Vries^1,2^, Pieternella J. Lugtenburg^4^, Sanne E. Wiegers^1,2^, Gerben J. C. Zwezerijnen^1,2^, Simone Pieplenbosch^2,3^, Lars Kurch^5^, Andreas Hüttmann^6^, Christine Hanoun^6^, Ulrich Dührsen^6^, Henrica C.W. de Vet^7,8^, PETRA*, Josée M. Zijlstra^2,3^ and Ronald Boellaard^1,2^

^1^Cancer Center Amsterdam, Department of Radiology and Nuclear Medicine, Amsterdam UMC, Vrije Universiteit Amsterdam, Amsterdam, Netherlands

^2^Cancer Center Amsterdam, Imaging and Biomarkers, Amsterdam, Netherlands

^3^Cancer Center Amsterdam, Department of Hematology, Amsterdam UMC, Vrije Universiteit Amsterdam, Amsterdam, Netherlands

^4^Department of Hematology, Erasmus MC Cancer Institute, University Medical Center Rotterdam, Rotterdam, The Netherlands

^5^Department of Nuclear Medicine, Clinic and Polyclinic for Nuclear Medicine, University of Leipzig, Leipzig, Germany

^6^Department of Hematology, West German Cancer Center, University Hospital Essen, University of Duisburg-Essen, Essen, Germany

^7^Department of Epidemiology and Data Science, Amsterdam Public Health Research Institute, Amsterdam UMC, Vrije Universiteit Amsterdam, Amsterdam, The Netherlands

^8^Department of Methodology, Amsterdam Public Health research institute, Methodology, Amsterdam, The Netherland

*A list of authors and their affiliations appears at the end of the paper

**Corresponding Author**: Maria C. Ferrández

[m.c.ferrandezferrandez@amsterdamumc.nl](mailto:m.c.ferrandezferrandez@amsterdamumc.nl)

Amsterdam UMC, De Boelelaan 1117, 1118,

1081 HV Amsterdam, The Netherlands

Tel: +31(0)204449638

Fax: +31(0)204444329

**PETRA CONSORTIUM**

Josée M. Zijlstra^1,2^, Ronald Boellaard^1,3^, Henrica C.W. de Vet^4,5^, Otto S. Hoekstra^1,2^, Jakoba J. Eertink^1,2^, Coreline N. Burggraaff^2^, Sanne E. Wiegers^1,3^, Simone Pieplenbosch^1,2^, Maria C. Ferrández^2,3^, Sandeep S. V. Golla^2,3^, Gerben J. C. Zwezerijnen^2,3^, Annelies Bes^2^, Martijn Heymans^6,7^, Yvonne W. S. Jauw^1,2^, Pieternella J. Lugtenburg^8^, Martine E. D. Chamuleau^2^, Sally F. Barrington^9^, George Mikhaeel^10^, Lars Kurch^11^, Andreas Hüttmann^12^, Christine Hanoun^12^, Ulrich Dührsen^12^, Emanuele Zuca^13,14^, Luca Ceriani^13,15^, Robert Carr^16^, Tamás Györke^17,18^, Sándor Czibor^19^, Stefano Fanti^20,21,22^, Lale Kostakoglu^23^, Annika Loft^24^, Martin Hutchings^25^ and Sze Ting Lee^26^

**Affiliations**

^1^Cancer Center Amsterdam, Imaging and Biomarkers, Amsterdam, Netherlands, ^2^Cancer Center Amsterdam, Department of Hematology, Amsterdam UMC, Vrije Universiteit Amsterdam, Amsterdam, Netherlands, ^3^Cancer Center Amsterdam, Department of Radiology and Nuclear Medicine, Amsterdam UMC, Vrije Universiteit Amsterdam, Amsterdam, Netherlands, *^4^*Department of Epidemiology and Data Science, Amsterdam Public Health Research Institute, Amsterdam UMC, Vrije Universiteit Amsterdam, Amsterdam, The Netherlands, *^5^*Department of Methodology, Amsterdam Public Health research institute, Methodology, Amsterdam, The Netherland, *^6^*Department of Epidemiology and Data Science, Amsterdam Public Health Research Institute, Amsterdam UMC, Vrije Universiteit Amsterdam, Amsterdam, The Netherlands, *^7^*Amsterdam Public Health research institute, Methodology, Amsterdam, The Netherland, ^8^Department of Hematology, Erasmus MC Cancer Institute, University Medical Center Rotterdam, Rotterdam, The Netherlands, ^9^King’s College London and Guy’s and St Thomas’ PET Centre, School of Biomedical Engineering and Imaging Sciences, King’s Health Partners, King’s College London, London, United Kingdom, ^10^Department of Clinical Oncology, Guy’s Cancer Centre and School of Cancer and Pharmaceutical Sciences, King’s College London University, London, United Kingdom, ^11^Department of Nuclear Medicine, Clinic and Polyclinic for Nuclear Medicine, University of Leipzig, Leipzig, Germany, ^12^Department of Hematology, West German Cancer Center, University Hospital Essen, University of Duisburg-Essen, Essen, Germany, ^13^SAKK Swiss Group for Clinical Cancer Research, Bern, Switzerland, ^14^Department of Oncology, IOSI - Oncology Institute of Southern Switzerland, Universita` della Svizzera Italiana, Bellinzona, Switzerland, ^15^Department of Nuclear Medicine and PET/CT Centre, Imaging Institute of Southern Switzerland, Universita` della Svizzera Italiana, Bellinzona, Switzerland, ^16^Guy’s and St. Thomas’ Hospital, King’s College, London, United Kingdom, ^17^Department of Nuclear Medicine, Semmelweis University, Budapest, Hungary, ^18^ScanoMed Medical Diagnostic Research and Training Ltd., Budapest, Hungary, ^19^Semmelweis University; Medical Imaging Centre, Department of Nuclear Medicine, ^20^Nuclear Medicine Unit, IRCCS Azienda Ospedaliero-Universitaria di Bologna, Bologna, Italy, ^21^Radiology Unit, IRCCS Azienda Ospedaliero-Universitaria di Bologna, Bologna, Italy, ^22^Nuclear Medicine, Alma Mater Studiorum, University of Bologna, Bologna, Italy, ^23^Department of Radiology and Medical Imaging, University of Virginia, Charlottesville,Virginia, USA, ^24^Department of Clinical Physiology and Nuclear Medicine, Rigshospitalet, 2100 Copenhagen, Denmark, ^25^Rigshospitalet, Copenhagen, Denmark, ^26^Australasian Association of Nuclear Medicine Specialists, Balmain, New South Wales, Australia

**Supplemental Material 1**

For BR-MIP CNN, the brain was removed from the MIPs during preprocessing. The CNN preprocessing tool (an in-house built tool), used to generate the MIPs from the 3D PET scans, located the brain exactly at the centre of the x axis (x = 100) for every image, which provides a reference value for the brain location to generate these new MIPs. An intensity profile was defined along the y axis at x = 100 from which the transition from brain to non-brain region (i.e. high intensity to low intensity) could be identified. This enabled the definition of a box which contained the brain segment for each image. Within this box, the *Otsu* threshold was calculated to generate a brain mask (*25*). As a final step, these masks were dilated and used to remove the brains from the images.

DLBCL patients might develop lesions nearby or within the brain region. To keep these lesions while removing the brain, the MIPs were thresholded at different SUV intensities generating a mask of the lesions. This mask was used to recover any partially truncated lesions during the process of removing the brain.

**Supplemental Material 2**

The loss and accuracy of the CNN were reported after every epoch using categorical cross entropy and categorical accuracy, respectively. The best model was saved for the epoch with the best performance, indicated by an increase in both training and validation accuracy or decrease in loss. This process was performed for each of the subsets A-E (Supplemental Figure 1) which were divided into training and internal validation sets (80% and 20% respectively) following a 5-fold cross validation. In addition, the confusion matrix with associated true positive (TP), true negative (TN), false positive (FP) and false negative (FN) predictions were retrieved for each fold. This allowed for the sensitivity (TP/ TP + FN) and specificity (TN / FP + TN) to be determined (6). Differences in sensitivity and specificity between models are compared at a probability threshold of 0.5.

**Supplemental Table 1.** Patients Characteristics.

| **HOVON-84** | | **PETAL** | |
| --- | --- | --- | --- |
| **Characteristic** | **N (%)** | **Characteristic** | **N (%)** |
| **Age**  Median (IQR)  ≤ 60 years  > 60 years | 65 (55-72)  97 (33)  199 (67) | **Age**  Median (IQR)  ≤ 60 years  > 60 years | 60 (50-69)  172 (51)  168 (49) |
| **Sex** Male  Female | 152 (51)  144 (49) | **Sex** Male  Female | 194 (57)  146 (43) |
| **Ann Arbor stage**  I  II  III  IV  missing | 0 (0)  48 (16)  62 (21)  186 (63)  0 (0) | **Ann Arbor stage**  I  II  III  IV  missing | 65 (19)  79 (23)  65 (19)  130 (38)  1 (1) |
| **LDH** normal  > normal  missing | 97 (33)  199 (67)  0 (0) | **LDH** normal  > normal  missing | 148 (44)  191 (55)  1 (1) |
| **Extranodal localizations** ≤1 >1  missing | 175 (59)  121 (41)  0 (0) | **Extranodal localizations** ≤1 >1  missing | 239 (70)  99 (29)  2 (1) |
| **WHO performance status** 0  1  2  3  missing | 170 (57)  87 (29)  37 (13)  0 (0)  2 (1) | **WHO performance status** 0  1  2  3  missing | 156 (46)  150 (44)  25 (7)  6 (2)  3 (1) |
| **IPI** Low  Low-intermediate  High-intermediate  High | 48 (16)  73 (25)  103 (35)  72 (24) | **IPI** Low  Low-intermediate  High-intermediate  High | 123 (36)  92 (27)  84 (25)  40 (12) |

*Abbreviations: IQR: Interquartile range, LDH: lactate dehydrogenase level, WHO: World Health Organization, IPI: International Prognostic Index*

**Supplemental Table 2**. Cross-validation (±SD) of AUC (training and internal validation), sensitivity and specificity for BR-MIP CNN.

|  | **CV-AUC** | | **Sensitivity** | | **Specificity** | |
| --- | --- | --- | --- | --- | --- | --- |
|  | Training | Validation | Training | Validation | Training | Validation |
| **Subset A** | 0.751 (0.035) | 0.684 (0.077) | 0.639 (0.057) | 0.656 (0.021) | 0.726 (0.051) | 0.689 (0.168) |
| **Subset B** | 0.759 (0.015) | 0.601 (0.081) | 0.759 (0.076) | 0.589 (0.197) | 0.610 (0.099) | 0.556 (0.178) |
| **Subset C** | 0.768 (0.086) | 0.717 (0.109) | 0.727 (0.174) | 0.710 (0.142) | 0.687 (0.11) | 0.629 (0.195) |
| **Subset D** | 0.749 (0.015) | 0.646 (0.137) | 0.768 (0.056) | 0.65 (0.157) | 0.591 (0.053) | 0.512 (0.172) |
| **Subset E** | 0.760 (0.066) | 0.626 (0.053) | 0.721 (0.176) | 0.65 (0.140) | 0.677 (0.127) | 0.621 (0.168) |

*Abbreviations: AUC: Area Under the Curve, SD: Standard Deviation*

**Supplemental Table 3**. Cross-validation (±SD) of AUC (training and internal validation), sensitivity and specificity for MIP CNN.

|  | **CV-AUC** | | **Sensitivity** | | **Specificity** | |
| --- | --- | --- | --- | --- | --- | --- |
|  | Training | Validation | Training | Validation | Training | Validation |
| **Subset A** | 0.751 (0.026) | 0.688 (0.066) | 0.653 (0.079) | 0.636 (0.24) | 0.706 (0.048) | 0.669 (0.122) |
| **Subset B** | 0.752 (0.014) | 0.648 (0.105) | 0.764 (0.077) | 0.767 (0.134) | 0.586 (0.092) | 0.556 (0.148) |
| **Subset C** | 0.792 (0.028) | 0.703 (0.062) | 0.759 (0.045) | 0.750 (0.084) | 0.639 (0.063) | 0.554 (0.102) |
| **Subset D** | 0.739 (0.015) | 0.665 (0.094) | 0.759 (0.059) | 0.727 (0.180) | 0.61 (0.120) | 0.501 (0.102) |
| **Subset E** | 0.761 (0.065) | 0.641 (0.115) | 0.682 (0.135) | 0.612 (0.129) | 0.716 (0.066) | 0.643 (0.12) |

*Abbreviations: AUC: Area Under the Curve, SD: Standard Deviation*

**Supplemental Table 4**. Cross-validation (±SD) of AUC (training and internal validation), sensitivity and specificity for lesion MIP CNN.

|  | **CV-AUC** | | **Sensitivity** | | **Specificity** | |
| --- | --- | --- | --- | --- | --- | --- |
|  | Training | Validation | Training | Validation | Training | Validation |
| **Subset A** | 0.731 (0.020) | 0.562 (0.094) | 0.624 (0.066) | 0.481 (0.098) | 0.692 (0.043) | 0.581 (0.239) |
| **Subset B** | 0.727 (0.028) | 0.616 (0.142) | 0.639 (0.056) | 0.610 (0.198) | 0.673 (0.045) | 0.596 (0.077) |
| **Subset C** | 0.807 (0.023) | 0.764 (0.07) | 0.696 (0.063) | 0.632 (0.195) | 0.764 (0.019) | 0.730 (0.076) |
| **Subset D** | 0.752 (0.039) | 0.646 (0.14) | 0.706 (0.032) | 0.650 (0.154) | 0.663 (0.113) | 0.556 (0.153) |
| **Subset E** | 0.775 (0.021) | 0.676 (0.060) | 0.706 (0.042) | 0.618 (0.105) | 0.754 (0.017) | 0.707 (0.116) |

*Abbreviations: AUC: Area Under the Curve, SD: Standard Deviation*


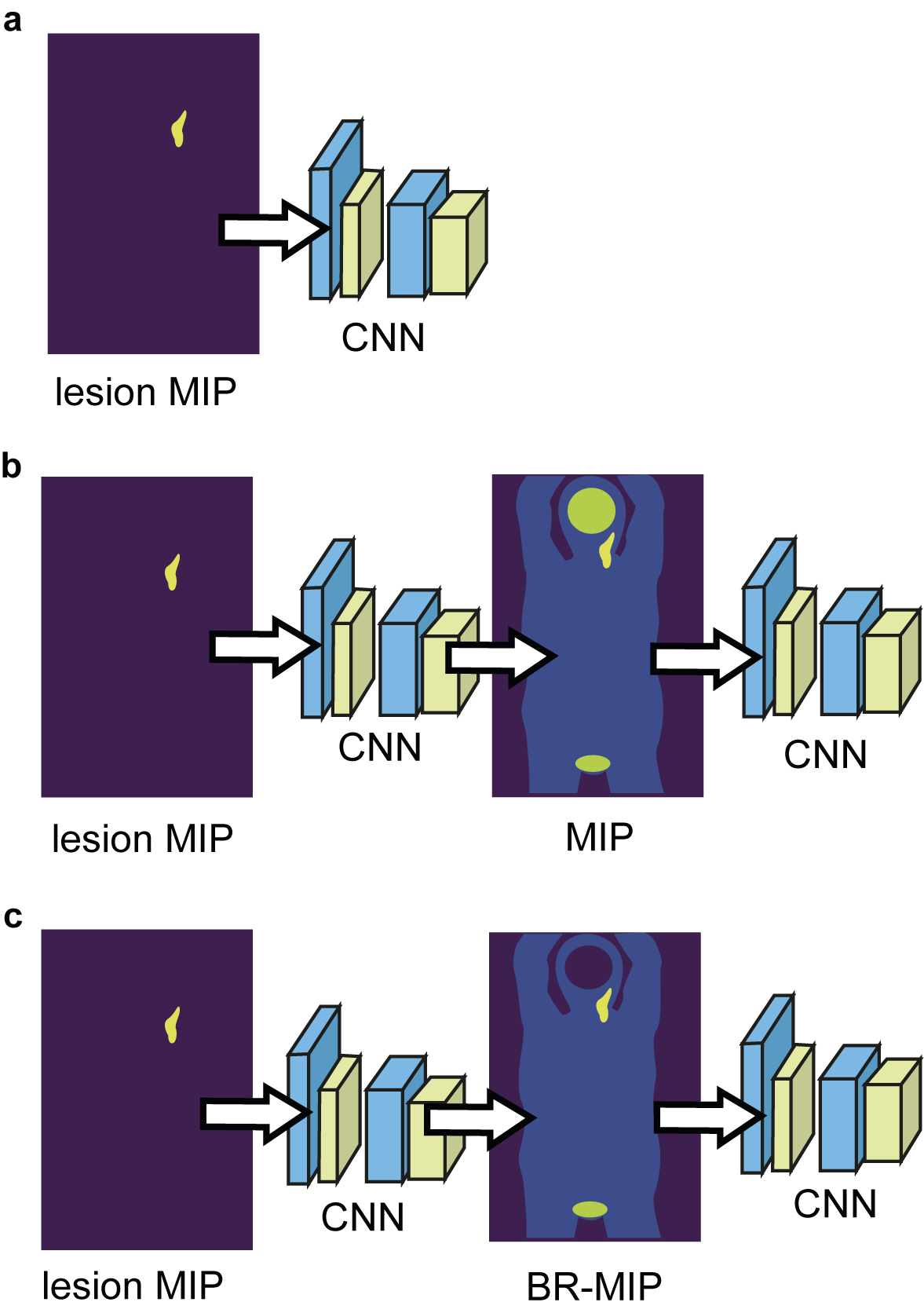


**Supplemental Figure 1**. Illustration of the three different training schemes implemented in this study (a) Lesion MIP CNN, (b) MIP CNN (c) BR-MIP CNN.


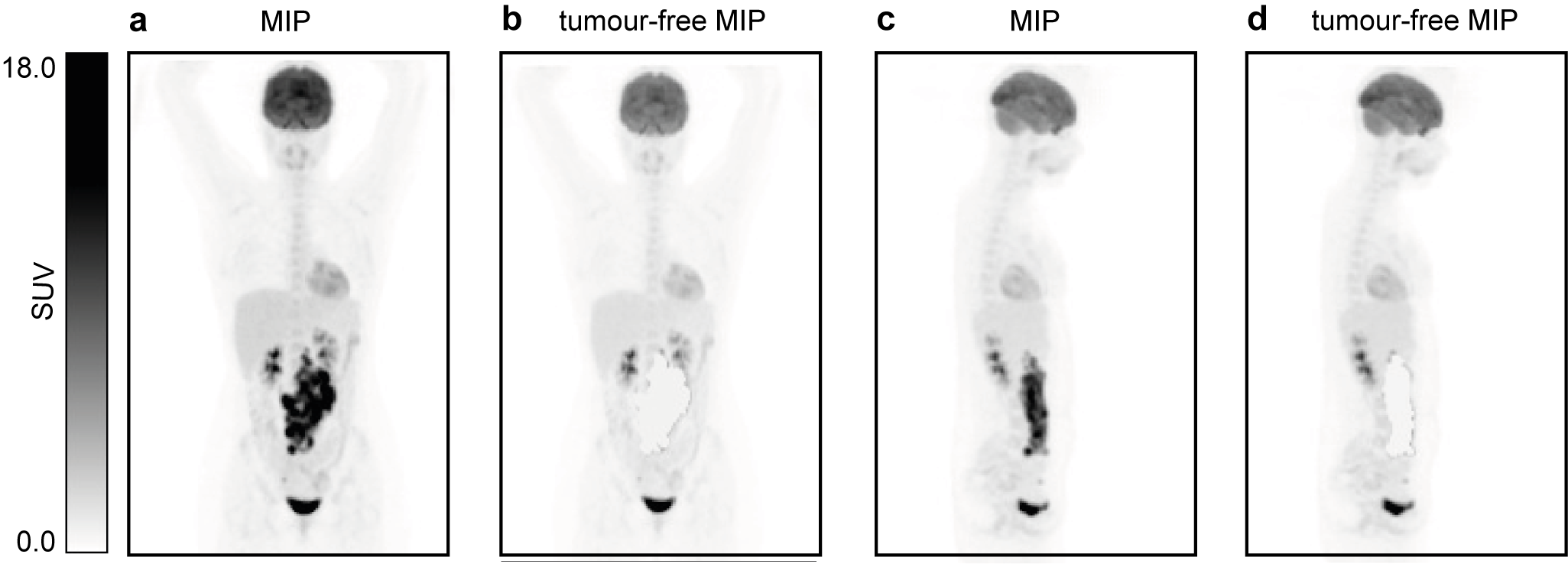


**Supplemental Figure 2**. Illustration of MIPs before and after removing the tumours. (A) Coronal view. (B) Sagittal view.


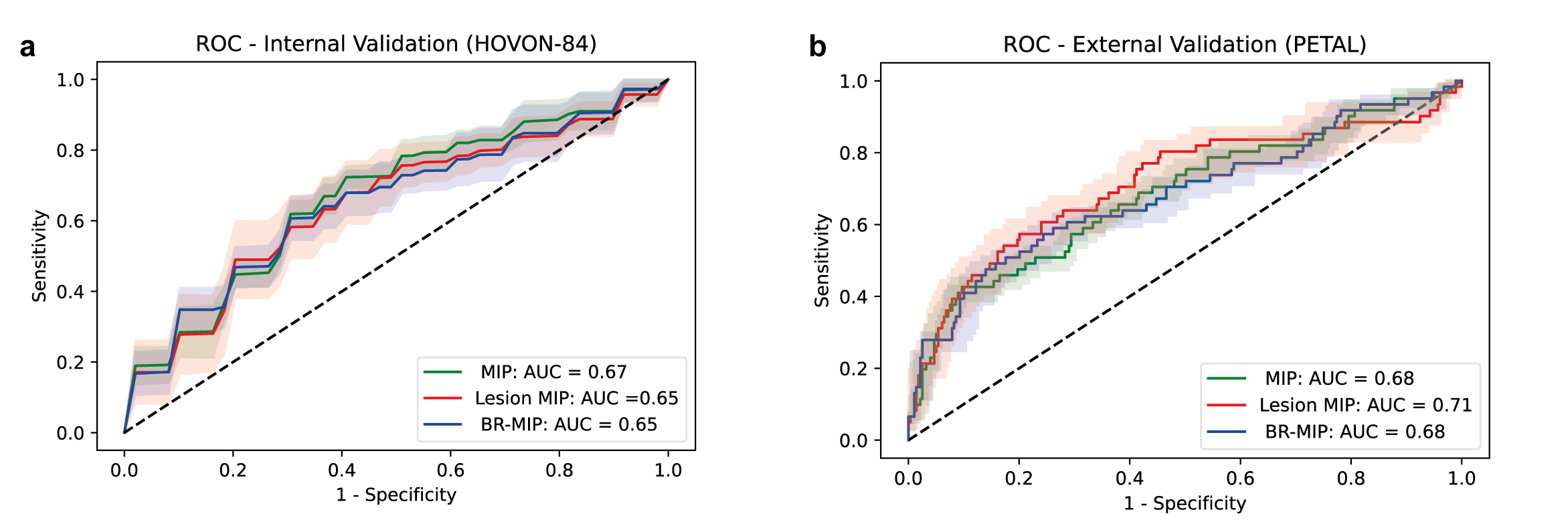


**Supplemental Figure 3**. Average Receiver Operator Curves (± standard deviation) for the models trained on the 5 subsets (A to E) for the 3 CNNs. (a) ROC and AUC for internal validation performed on HOVON-84 dataset. (b) ROC and AUC for external validation performed on PETAL dataset. Standard deviations associated with the 5 subsets (A to E) are shown in faded colours around the AUC curves. For reference, a model without any predictive performance is depicted (AUC = 0.5).


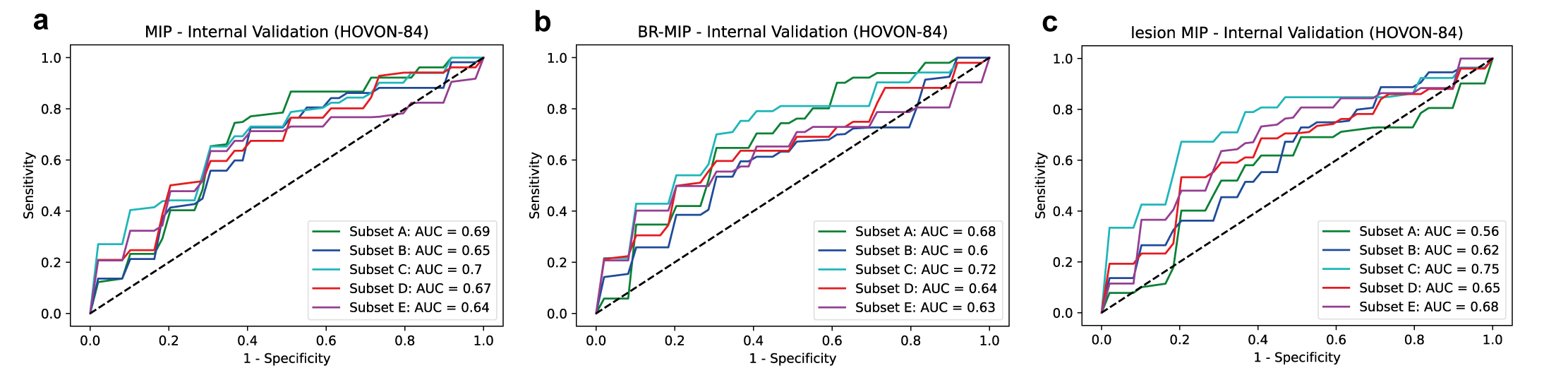


**Supplemental Figure 4**. Receiver Operator Curves for all models trained on subsets A to E for internal validation performed on HOVON-84 dataset. (a) MIP CNN (b) BR-MIP CNN (c) Lesion MIP CNN.


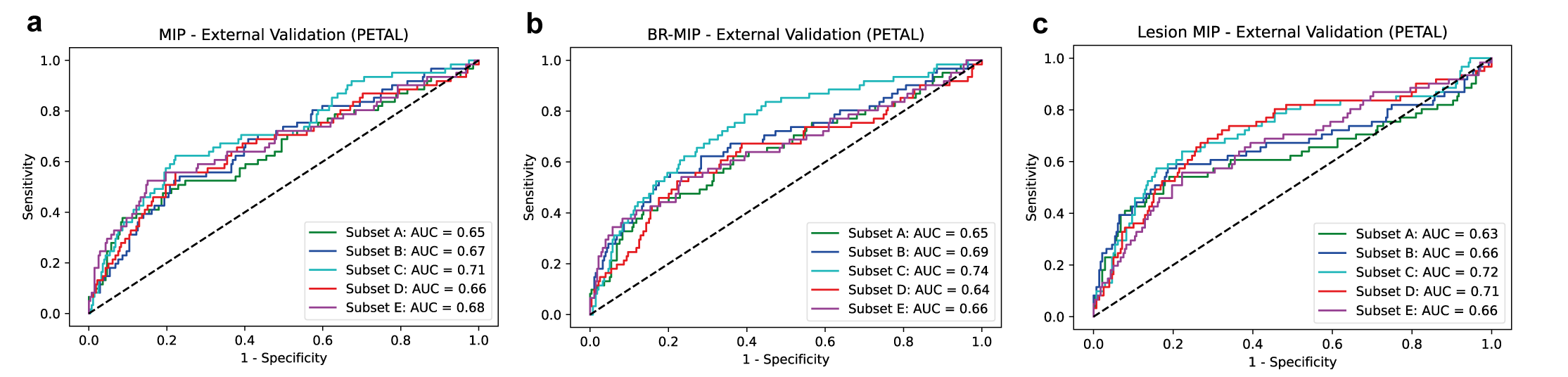


**Supplemental Figure 5**. Receiver Operator Curves for all models trained on subsets A to E for external validation performed on PETAL dataset. (a) MIP CNN (b) BR-MIP CNN (c) Lesion MIP CNN.


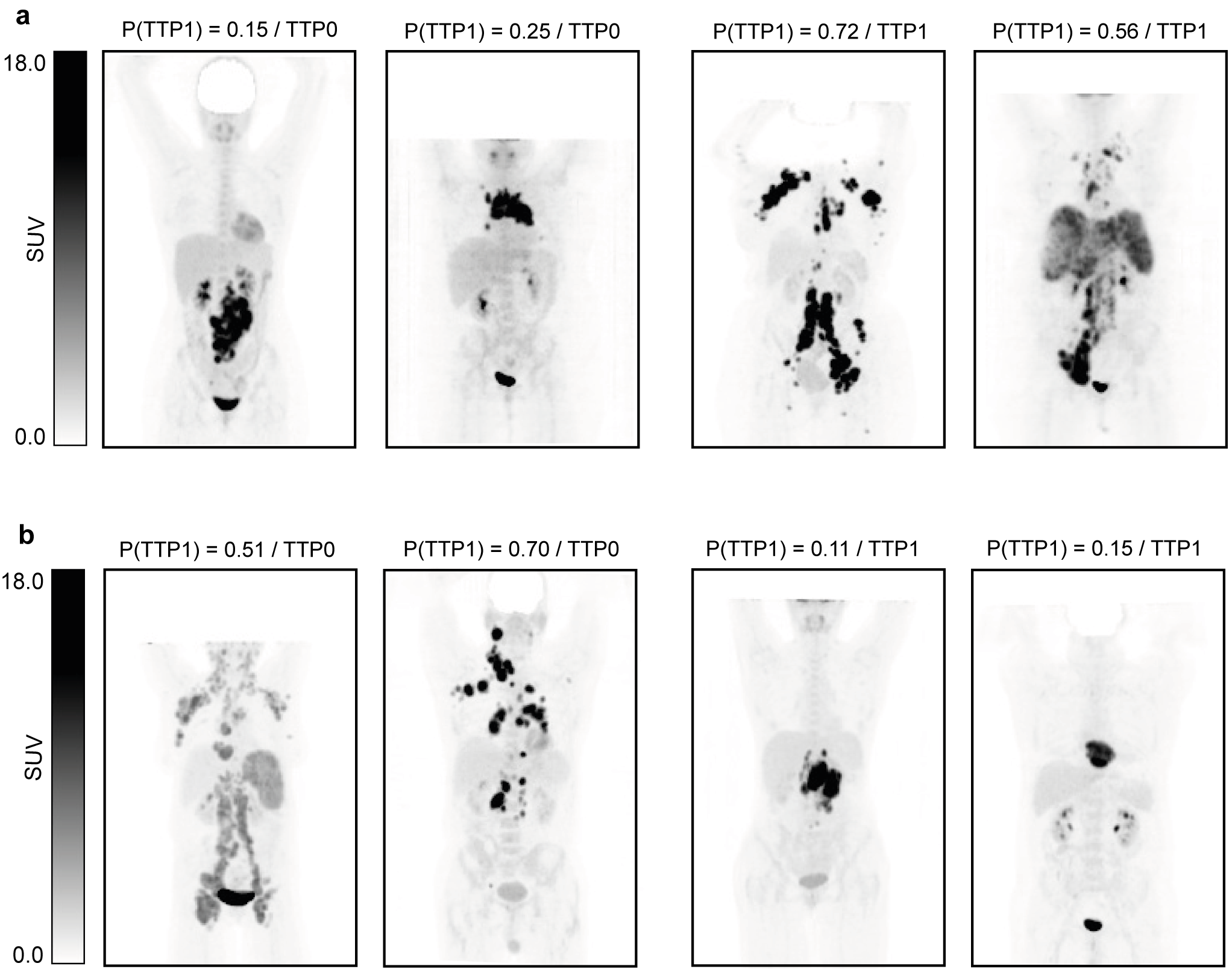


**Supplemental Figure 6**. BR-MIP CNN predictions. (a) Four patients which were correctly classified by the model trained and cross-validated on subset C. (b) Four examples which were wrongly classified by the model. P(TTP1) indicates the CNN calibrated probabilities and /TTP0 or /TTP1 indicates the original label.


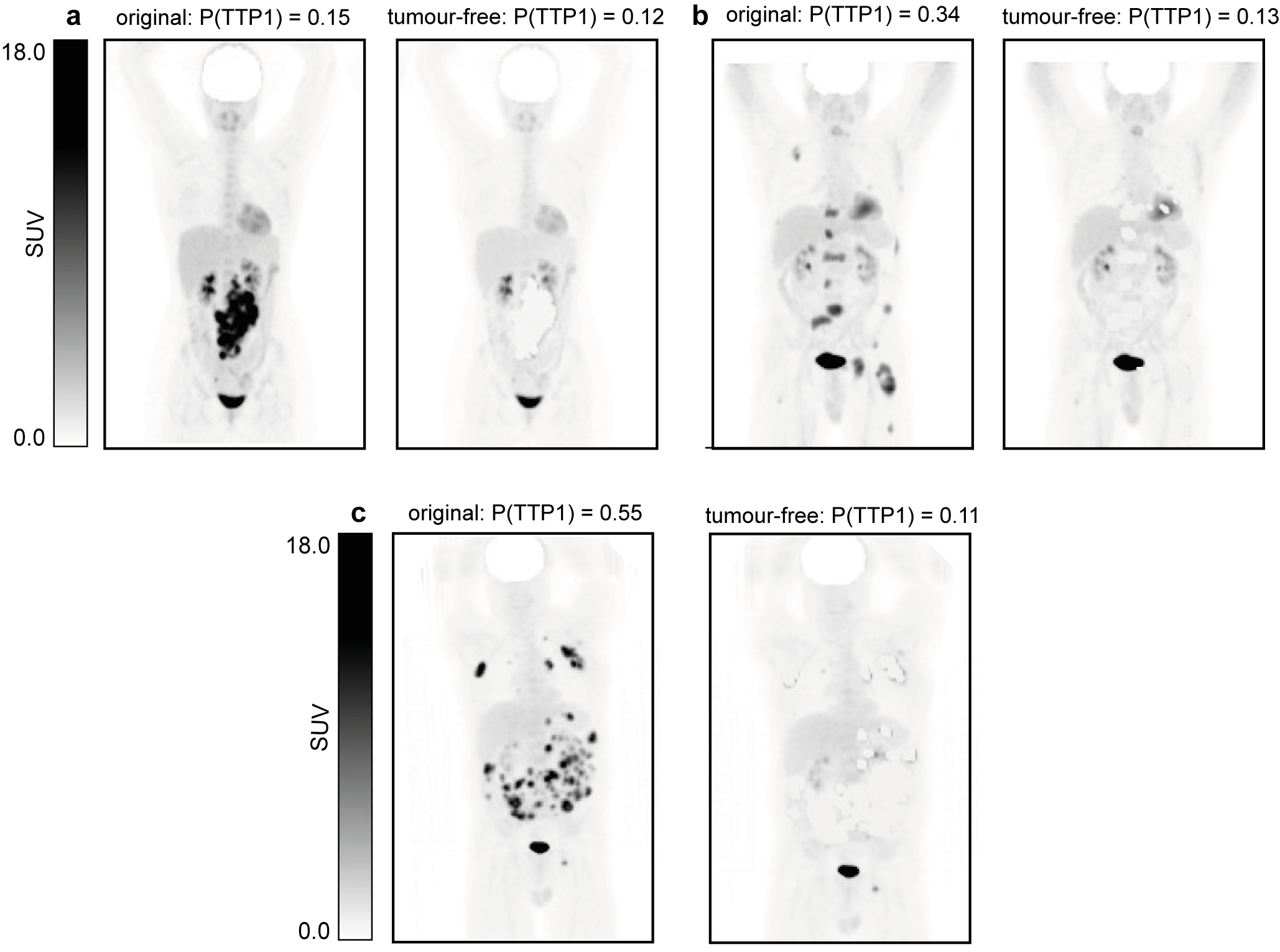


**Supplemental Figure 7**. TTP1 calibrated probabilities before and after removing the tumours. Figures (a) to (c) show 3 different patients before and after removing (‘ablating’) the tumours (i.e. tumour-free MIPs).
